# Supplementary material for: Diversity, Pattern, and Environmental Drivers of Climbing Plants in China
Source: Plants (Basel). 2025 Oct 27;14(21):3281. doi: 10.3390/plants14213281 (PMC12608777; doi:10.3390/plants14213281)
Supplement: Supplementary file 1 [file plants-14-03281-s001.zip › Table S1. Species richness and endemism of Chinese climbing plants.pdf]

Table S1. Species richness and endemism of Chinese climbing plants.

Family name	No. of Genus	Species		Species endemic to China	
		No.	Percentage (%)	No.	Percentage (%)
Fabaceae	88	352	10.10	153	4.39
Apocynaceae	59	338	9.70	179	5.14
Ranunculaceae	4	229	6.57	157	4.51
Rosaceae	3	209	6.00	153	4.39
Vitaceae	11	187	5.37	129	3.70
Cucurbitaceae	35	185	5.31	101	2.90
Convolvulaceae	21	146	4.19	47	1.35
Rubiaceae	19	146	4.19	79	2.27
Aristolochiaceae	2	101	2.90	82	2.35
Smilacaceae	1	88	2.53	46	1.32
Menispermaceae	19	84	2.41	48	1.38
Actinidiaceae	2	77	2.21	69	1.98
Celastraceae	12	76	2.18	50	1.43
Rhamnaceae	10	67	1.92	37	1.06
Dioscoreaceae	1	62	1.78	29	0.83
Piperaceae	1	61	1.75	38	1.09
Annonaceae	7	48	1.38	18	0.52
Oleaceae	4	47	1.35	23	0.66
Lardizabalaceae	7	39	1.12	27	0.77
Moraceae	6	39	1.12	14	0.40
Asteraceae	18	35	1.00	17	0.49
Lamiaceae	13	35	1.00	15	0.43
Gentianaceae	3	34	0.98	24	0.69
Arecaceae	5	32	0.92	15	0.43
Schisandraceae	2	32	0.92	20	0.57
Campanulaceae	5	31	0.89	12	0.34
Capparaceae	1	31	0.89	10	0.29
Primulaceae	4	28	0.80	9	0.26
Rutaceae	6	27	0.77	15	0.43
Elaeagnaceae	1	26	0.75	21	0.60
Malpighiaceae	4	25	0.72	14	0.40
Araceae	7	24	0.69	2	0.06
Caprifoliaceae	2	24	0.69	13	0.37
Passifloraceae	2	23	0.66	7	0.20
Poaceae	7	23	0.66	18	0.52
Acanthaceae	5	22	0.63	10	0.29
Commelinaceae	7	22	0.63	5	0.14

Sabiaceae	1	22	0.63	12	0.34
Gesneriaceae	2	19	0.55	11	0.32
Hydrangeaceae	5	19	0.55	16	0.46
Loganiaceae	4	19	0.55	3	0.09
Hernandiaceae	1	18	0.52	8	0.23
Araliaceae	5	17	0.49	8	0.23
Salicaceae	1	17	0.49	13	0.37
Plantaginaceae	7	16	0.46	8	0.23
Asparagaceae	2	15	0.43	8	0.23
Euphorbiaceae	7	14	0.40	8	0.23
Combretaceae	2	13	0.37	2	0.06
Orchidaceae	7	13	0.37	6	0.17
Orobanchaceae	4	13	0.37	9	0.26
Urticaceae	7	13	0.37	1	0.03
Malvaceae	4	11	0.32	4	0.11
Melastomataceae	4	11	0.32	5	0.14
Papaveraceae	2	11	0.32	3	0.09
Polygonaceae	4	11	0.32	7	0.20
Gnetaceae	1	10	0.29	7	0.20
Solanaceae	2	10	0.29	2	0.06
Connaraceae	6	9	0.26	1	0.03
Icacinaceae	6	9	0.26	2	0.06
Lygodiaceae	1	9	0.26	0	0.00
Linderniaceae	2	8	0.23	3	0.09
Phrymaceae	2	7	0.20	3	0.09
Santalaceae	1	7	0.20	0	0.00
Resedaceae	1	6	0.17	3	0.09
Stemonaceae	1	6	0.17	3	0.09
Polygalaceae	2	5	0.14	3	0.09
Anacardiaceae	2	4	0.11	1	0.03
Cactaceae	4	4	0.11	0	0.00
Onagraceae	1	4	0.11	1	0.03
Basellaceae	2	3	0.09	0	0.00
Bignoniaceae	2	3	0.09	1	0.03
Boraginaceae	2	3	0.09	0	0.00
Cornaceae	2	3	0.09	1	0.03
Grossulariaceae	1	3	0.09	2	0.06
Hypericaceae	1	3	0.09	1	0.03
Nyctaginaceae	2	3	0.09	0	0.00
Olacaceae	2	3	0.09	1	0.03
Verbenaceae	3	3	0.09	0	0.00
Amaranthaceae	2	2	0.06	0	0.00
Buxaceae	1	2	0.06	2	0.06
Cannabaceae	1	2	0.06	1	0.03

Dilleniaceae	1	2	0.06	0	0.00
Opiliaceae	2	2	0.06	0	0.00
Plumbaginaceae	1	2	0.06	1	0.03
Ancistrocladaceae	1	1	0.03	0	0.00
Blechnaceae	1	1	0.03	0	0.00
Cardiopteridaceae	1	1	0.03	0	0.00
Dichapetalaceae	1	1	0.03	0	0.00
Droseraceae	1	1	0.03	0	0.00
Ericaceae	1	1	0.03	1	0.03
Flagellariaceae	1	1	0.03	0	0.00
Gelsemiaceae	1	1	0.03	0	0.00
Lauraceae	1	1	0.03	0	0.00
Liliaceae	1	1	0.03	1	0.03
Lycopodiaceae	1	1	0.03	0	0.00
Mazaceae	1	1	0.03	1	0.03
Nepenthaceae	1	1	0.03	0	0.00
Pandanaceae	1	1	0.03	0	0.00
Phyllanthaceae	1	1	0.03	0	0.00
Salvadoraceae	1	1	0.03	0	0.00
Sapindaceae	1	1	0.03	0	0.00
Scrophulariaceae	1	1	0.03	0	0.00
Stachyuraceae	1	1	0.03	0	0.00
Tamaricaceae	1	1	0.03	0	0.00
Tropaeolaceae	1	1	0.03	0	0.00
**Total**	**551**	**3485**	**100.00**	**1880**	**53.95**
