# Supplementary material for: Diversity, Pattern, and Environmental Drivers of Climbing Plants in China
Source: Plants (Basel). 2025 Oct 27;14(21):3281. doi: 10.3390/plants14213281 (PMC12608777; doi:10.3390/plants14213281)
Supplement: Supplementary file 1 [file plants-14-03281-s001.zip › Table S2. Grouping and screening of 19 climatic factors, the screening criterion is the maximum correlation coefficient (r) of each group.pdf]

Table S2. Grouping and screening of 19 climatic factors, the screening criterion is the maximum correlation coefficient (r) of each group.

Groups	Climatic factors	Climbing plants	Evergreen woody liana	Deciduous woody liana	Herbaceous vine
**Group 1**	Bio3	√	√	√	√
**Group 2**	Bio4				
	Bio7	√	√	√	√
**Group 3**	Bio1				
	Bio6				
	Bio9				
	Bio11	√	√	√	√
**Group 4**	Bio12			√	
	Bio13				
	Bio16	√	√		
	Bio18				√
**Group 5**	Bio2	√	√	√	√
**Group 6**	Bio14	√	√	√	√
	Bio17				
	Bio19				
**Group 7**	Bio15	√	√	√	√
**Group 8**	Bio5				
	Bio8				√
	Bio10	√	√	√	
