# Supplementary material for: Diversity, Pattern, and Environmental Drivers of Climbing Plants in China
Source: Plants (Basel). 2025 Oct 27;14(21):3281. doi: 10.3390/plants14213281 (PMC12608777; doi:10.3390/plants14213281)
Supplement: Supplementary file 1 [file plants-14-03281-s001.zip › Table S3. The corrected species densities of entire, endemic, threatened and invasive climbing plants across the 28 geographical units in China.pdf]

Table S3. The corrected species densities of entire, endemic, threatened and invasive climbing plants across the 28 geographical units in China.

**Province**	**Climbing plants species density**	**Endemic plants species density**	**Threatened plants species density**	**Invasive plants species density**
Anhui	256.45	107.29	20.93	2.62
Fujian	480.14	186.57	39.33	8.23
Gansu	160.19	73.04	8.59	1.23
Guangdong	721.17	271.13	64.40	10.34
Guangxi	917.98	394.67	116.29	5.09
Guizhou	716.69	345.91	65.01	2.41
Hainan	1182.14	338.80	169.40	18.21
Hebei	97.99	23.94	2.24	2.24
Heilongjiang	51.35	5.37	2.39	0.60
Henan	177.47	79.33	10.63	1.64
Hubei	398.65	225.32	27.57	1.58
Hunan	431.40	221.73	38.46	3.02
Jiangsu	201.90	61.61	9.48	4.74
Jiangxi	362.39	169.33	31.90	3.27
Jilin	74.64	9.43	4.71	0.79
Liaoning	88.71	11.09	3.41	0.85
Nei Mongol	34.73	4.82	0.48	1.45
Ningxia	58.38	13.38	0.00	2.43
Qinghai	32.82	7.53	1.08	0.54
Shaanxi	226.96	111.96	11.42	3.05
Shandong	91.40	18.45	2.52	2.52
Shanxi	110.45	36.82	4.18	1.67
Sichuan	512.35	296.35	51.86	1.71
Taiwan	950.92	284.02	93.47	21.57
Xinjiang	32.41	4.95	1.80	1.80
Xizang	208.63	62.01	20.67	0.96
Yunnan	1217.09	559.04	201.80	5.01
Zhejiang	427.07	200.34	39.09	7.82
