# Supplementary figures and images for: Diversity, Pattern, and Environmental Drivers of Climbing Plants in China
Source: Plants (Basel). 2025 Oct 27;14(21):3281. doi: 10.3390/plants14213281 (PMC12608777; doi:10.3390/plants14213281)

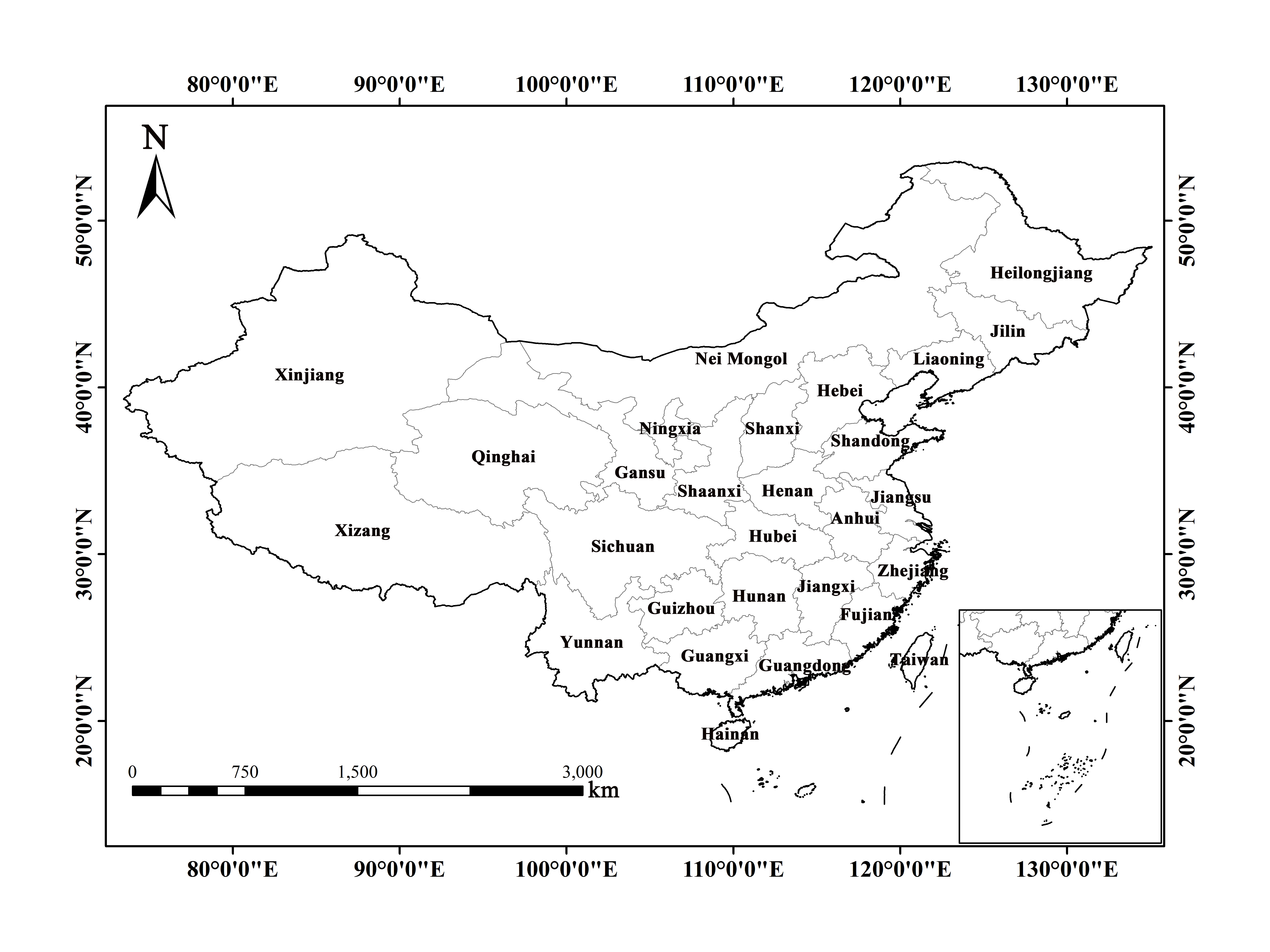

Supplement: Supplementary file 1 [file plants-14-03281-s001.zip › Figure S1. The 28 geographical units of China in this study.jpg]
